# A genome-wide approach to identify genetic loci with a signature of natural selection in the Irish population

**DOI:** 10.1186/gb-2006-7-8-r74

**Published:** 2006-08-11

**Authors:** Valeria Mattiangeli, Anthony W Ryan, Ross McManus, Daniel G Bradley

**Affiliations:** 1 Smurfit Institute of Genetics, Trinity College, Dublin 2, Ireland; 2 Department of Clinical Medicine, Trinity Centre for Health Science; Institute of Molecular Medicine, Dublin Molecular Medicine Centre, St James's Hospital, Dublin, Ireland; 3 Trinity College, Dublin, Ireland

## Abstract

**Background:**

In this study we present a single population test (Ewens-Waterson) applied in a genomic context to investigate the presence of recent positive selection in the Irish population. The Irish population is an interesting focus for the investigation of recent selection since several lines of evidence suggest that it may have a relatively undisturbed genetic heritage.

**Results:**

We first identified outlier single nucleotide polymorphisms (SNPs), from previously published genome-wide data, with high F_ST _branch specification in a European-American population. Eight of these were chosen for further analysis. Evidence for selective history was assessed using the Ewens-Watterson's statistic calculated using Irish genotypes of microsatellites flanking the eight outlier SNPs. Evidence suggestive of selection was detected in three of these by comparison with a population-specific genome-wide empirical distribution of the Ewens-Watterson's statistic.

**Conclusion:**

The cystic fibrosis gene, a disease that has a world maximum frequency in Ireland, was among the genes showing evidence of selection. In addition to the demonstrated utility in detecting a signature of natural selection, this approach has the particular advantage of speed. It also illustrates concordance between results drawn from alternative methods implemented in different populations.

## Abstract 2

A single population test applied in a genomic context reveals evidence of selection on three biologically interesting genes in the Irish population.

## Background

Ireland is an island on the western edge of Europe and genetic evidence suggests that its population history may have been relatively (but not absolutely) undisturbed by secondary migrations [1,2]. This genetic heritage could mean that population genetic signals, such as signatures of recent selection, are more readily detectable in the Irish than in other European populations. Furthermore, Ireland has world frequency extremes (or near extremes) for many variants that are suspected of having undergone selection, including disease-related genes, for example: the cystic fibrosis locus, *CFTR *[3,4]; the ABO blood group and the rhesus blood factor [5]; *GALT*, associated with galactosemia [6]; *HFE*, associated with haemochromatosis [7]; and *PKU*, associated with phenyketonuria [8].

The human genome sequence has provided a resource with enormous medical potential. However, we are as yet ignorant of the majority of genes that are medically important, especially with reference to common diseases, and the variations within those genes that matter. Knowledge of past selection may inform on these key points.

### Inference of selection from population genetics

One way to infer evidence of past selection is to compare variation in allele frequency at different loci among different populations. This assumes that geographically variable selective forces favor different variants in different regions. Hence, between-population allele frequency differences may be more extreme in genome portions harboring such variants. An established approach to detecting such genome regions is that of comparing F_ST _values among loci; F_ST _provides an estimate of how much genetic variability partitions between, rather than within, populations.

A particularly promising approach is that of population genomics. Here, the testing of large numbers of loci enables the compiling of an empirical distribution for a summary statistic, such as F_ST_, from which outlying values may be identified as biologically interesting [9,10]. An empirical approach may confer an additional level of rigor as model-based statistical tests may be sensitive to demographic effects that can produce allele frequency patterns similar to those seen under the presence of selection [11-16]. Importantly, general population demographic history will affect the whole genome but selection will only act on specific loci and these will show unusual deviation from genomic patterns. Human population-specific skews in these distributions have been previously demonstrated. For example, Tajima's *D *values in humans tend to be skewed towards negative values and this can be attributed to population expansion that occurred to humans post migration from Africa [17].

In an early genome-level analysis, analyzed 26,530 single nucleotide polymorphisms (SNPs) from 'The SNP Consortium' (TSC) allele frequency project in three populations (African-American, East Asian and European-American). From this three-way comparison, they identified 174 genes associated with SNPs whose extreme deviations in F_ST _values suggested histories of selection. Interestingly, two of these had been implicated in previous studies but another 18 were putative bioinformatically predicted candidate genes. The markers used in this work had been initially discovered by assaying a small number of chromosomes, leading to possible bias toward more common polymorphisms. It should also be noted that these and other results from genome scans are subject to problems arising from multiple testing and the 'winners curse' phenomenon [9]. Therefore, there is a clear and acknowledged need for follow-up analyses to verify the preliminary signatures of selection within this first generation selection map of the human genome.

Here we select, using an analysis of locus-specific branch lengths generated from the data of Akey and colleagues [12], eight SNPs that are good candidates to have undergone selection within European populations. We identify microsatellite markers flanking these loci, and genotype these in an Irish population sample that has previously been genotyped at 372 micrsosatellites throughout the whole genome. This allows a comparison between our test markers and an empirical distribution of the Ewans-Watterson statistic, a summary of within-population allele frequency spectra that is a test for selection. This demonstrates that seven of the SNPs are in the proximity of microsatellite markers with extreme frequency spectra, allowing stronger inference of selective history in north-western Europe at three biologically interesting genes.

## Results

### Locus specific branch length analysis

**Figure 1:**
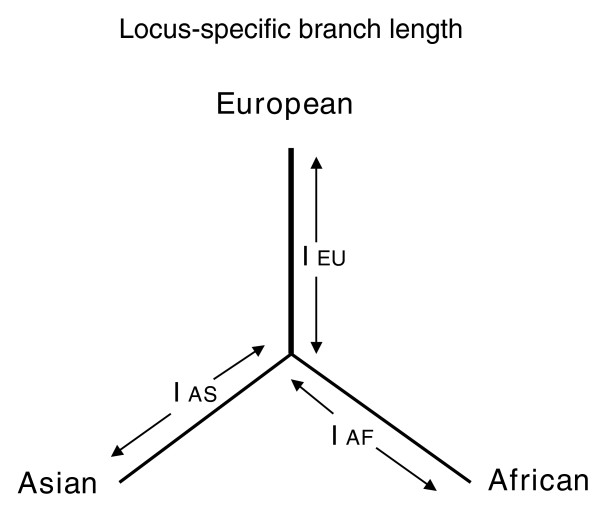
Schematic illustrating the use of pairwise F_ST _scores to generate a locus-specific branch length (as described in [18]). Branch lengths (l_EU_, l_AF_, l_AS_) were calculated from single locus pairwise F_ST _distances. l_EU _= (European:Asian F_ST _+ European-African F_ST _- Asian-African F_ST_)/2; l_AS _= (European-Asian F_ST _+ Asian-African F_ST _- European-African F_ST_)/2; l_AF _= (European-African F_ST _+ Asian-African F_ST _- European-Asian F_ST_)/2.

**Figure 2:**
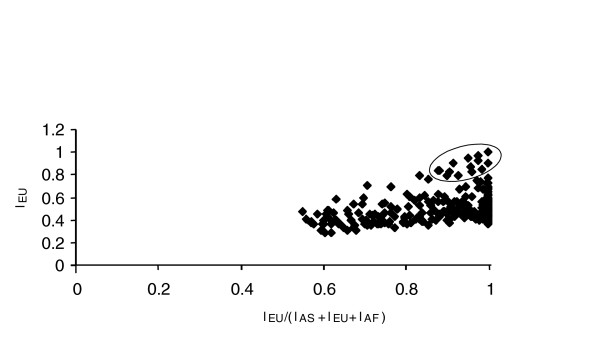
Absolute European locus-specific branch length (LSBL) plotted versus the relative LSBL for each SNP with significant F_ST _(> 0.45); *n *= 334. Data were from Akey and colleagues [12]. The 23 loci circled in the plot have an EU Absolute LSBL value = 0.8 and were considered in this study as outliers. EU, European-Americans; AF, African-Americans; AS, East Asian.

In the present study, we first selected SNPs with extreme F_ST _values from Akey and colleagues' [12] original data set of 26,530 (among all populations; F_ST _> 0.45; 812 SNPs selected from the upper 3% tail). The diversity at each SNP may be summarized in more detail by a simple three-branch phylogeny constructed from pairwise F_ST _distances. We focused on the locus-specific branch length (LSBL) from the European sample node to the central node in the network; an indication of differentiation that may be peculiar to Europe (Figure 1). This is an approach that has also been used by Shriver and colleagues [18] on a separate but similar data set. Values from the high tail of this statistic are considered in Figure 2, where the locus-specific European branch length (the absolute value) is plotted versus this statistic scaled by dividing it by the total locus-specific branch length. From this, 23 outlying SNPs showing a locus-specific branch length > 0.8 for the European population were examined. Among these, six were in known genes, six were in genes of unknown function and 11 were not in coding sequences. However, three of the latter were in proximity (within 50 kb) to a gene. A subset of eight SNPs (from the above 23), all the six SNPs in known genes, one in proximity to a gene and one not in coding sequences, were further investigated.

### Microsatellite diversity at proximal markers

Loci within a genomic region containing a selected variant may share the population genetic effects of its selection because of genetic hitchhiking. Thus, in an effort to verify the evidence for European-centerd selection we identified and investigated the population genetics of microsatellite markers flanking the eight SNPs described above. We genotyped these in an Irish test population and compared their diversity to that of 372 microsatellite markers for which there is genotype data from the same subjects. Fourteen microsatellites markers were developed during this project while two were taken from previously published literature (IVS8CA [19]; IVS17bTA [20]) (Table 1).

Two microsatellites proved impossible to genotype due to poor amplification and/or high levels of stuttering. All but one of the microsatellites genotyped did not deviate significantly from Hardy-Weinberg expectations (1 result of *p *= 0.04 in 15 tests; Table 1), indicating a population in equilibrium and supporting genotyping accuracy [21].

All microsatellites flanking the same SNP (Table 1) were tested for linkage disequilibrium. With the exception of microsatellites *TPSG-1 *versus *TPSG-2*, *IVS8CA *versus *IVS17bTA *and *IVS17bTA *versus *CFTR-3*, all the other linked markers showed significant values (*CFTR-3 *versus *IVS8CA*, *p *< 0.001; *TOX-1 *versus *TOX-2*, *p *= 0.0017; *ng-1 *versus *ng-2*, *p *= 0.045; *SYT9-1 *versus *SYT9-2*, *p *= 0.015; *PRKCH-1 *versus *PRKCH-2*, *p *< 0.001)

### Statistical tests to identify the signature of selection

In the present analysis putative signatures of selection were assessed in two different ways. Firstly, all the genotypes from the microsatellites flanking the eight outlier SNPs were used for a single population statistical test based on simulated values; a development of the Ewens-Watterson test implemented using the program BOTTLENECK. [22]. Secondly, divergences from the heterozygosity values expected under Ewens-Watterson were assessed against an empirical distribution of the same statistic calculated from 372 microsatellites, spread through the entire genome, genotyped on the same panel of individuals.

According to Ewens [23], under neutrality an expected configuration of allele counts can be calculated from the sample size and the observed number of alleles. Subsequently, a test (Ewens-Watterson) that compares the observed and expected allele configuration to determine departure from neutrality was developed [24]. Changes in allele proportions can be an indication of selection pressure. The differences in allele frequency spectra between that expected under neutrality and that observed can be quantified by the difference between observed gene diversity (equivalent to the Hardy-Weinberg expected heterozygosity) and expected Ewens-Watterson heterozygosity (a DH value) [22]. Each DH value is divided by the standard deviation (sd) of the gene diversity to standardize differences between microsatellite loci (DH/sd) and a significance value is assigned to DH/sd using simulations [22].

**Figure 3:**
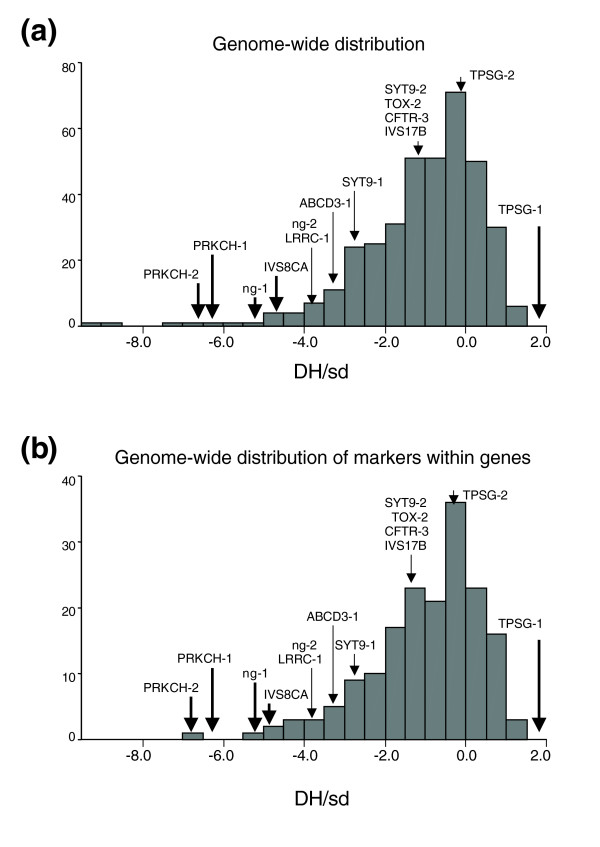
Empirical distribution of Ewens-Waterson's statistic. **(a) **Genome-wide distribution of Ewens-Watterson's analysis statistic (DH/sd) generated from 372 dinuclotide microsatellites, distributed through the whole genome (ABI PRISM Linkage mapping Set-MD10). **(b) **Genome-wide distribution of Ewens-Watterson's statistic generated from microsatellites located within genes. The number of loci is indicated by the Y-axis. The arrows show the Ewens-Watterson score for each microsatellite associated with the genes included in the preliminary test. The microsatellites in bold have significant divergence (P_E_, Table 1) between observed gene diversity and expected heterozygosity according to the Ewens-Watterson test.

Nine of the fourteen markers genotyped gave significant deviation from expected heterozygosity as assessed using the simulation test (Table 1). When the values (DH/sd) from the Ewens-Watterson test were compared against the genome-wide empirical distribution (Figure 3), all the microsatellites that had a significant DH/sd from simulation (P_S_, Table 1) were found in the tails (8%) of the distribution. Eight were in the negative and one in the positive tail; perhaps indicating different modes of selection. Only four microsatellites in the positive tail and one in the negative one had a significant DH/sd when more stringent significance values were calculated from the empirical distribution (P_E_, Table 1). The negative tail could indicate that the deviation between observed gene diversity and expected Ewens-Watterson heterozygosity is due to positive selection. This is because a selective sweep will lead to a high frequency of the selected allele, a reduction of variability and an excess of rare variants [10]. Therefore, a lower number of heterozygotes than expected are observed. On the other hand, the positive tail could indicate the presence of balancing selection where two or more alleles are maintained at higher frequency, leading to a higher number of observed heterozygotes. Furthermore, the Irish genome-wide empirical distribution is skewed towards negative values (Figure 3), a result consistent with the distribution across 5,257 microsatellites in individuals of European ancestry [13].

### The effects of proximity to coding sequences

We also examined the possibility that the tendency of the microsatellite test markers' DH/sd values to occur in the negative tail of the empirical distribution could be a consequence of bias due to their proximity to coding sequences and, thus, the effects of purifying selection. We constructed a sub-distribution of the empirical null by selecting only those markers that were within genes (introns or exons, *n *= 174) (Figure 3b). Clearly, a subset of the test markers remain in the tail of this more conservative distribution. A further indication of the neutrality of the gene-associated marker scores from the empirical distribution was that DH/sd values for these microsatellites did not correlate with their distances to the nearest gene (Spearman's rho = 0.08; Pearson correlation = 0.085).

### Correlation between divergence and allele frequency spectra

Many methods of assessing non-neutrality using population genetic data are known to be related and thus largely redundant. However, the two employed here belong to two separate approaches: locus-specific F_ST _branch length (LBSL) is based on divergence between populations and DH/sd is an assessment of allele frequency spectra within a single group. To empirically assess the potential complementarity of these approaches, we took a data set composed of 377 microsatellite markers typed in French, Han Chinese and Nigerian Yoruba samples [25]. We calculated two statistics analagous to those we used above: DH/sd in each sample plus the LSBL terminating with the same group. In the European (French) sample the two were negatively correlated (Spearman's Rank correlation: r_s _= -0.233; *p *< 0.001). However, LSBL proves a poor predictor of DH/sd; for example, when one examines the top 5% of LSBL score outliers, only 1/19 is also a 5% outlier for DH/sd. Analysis of the rank correlation between LBSL and DH/sd in the Yoruba sample gave a weaker correlation result (r_s _= -0.155; *p *< 0.003) and in the Han sample a somewhat stronger correspondence (r_s _= 0.308; *p *< 0.001). In the former and the latter, respectively, 3/19 and 5/19 of the top 5% outliers coincided for the two approaches.

## Discussion

We have found population genetic evidence suggestive of a signature of selection at several markers associated with genes in an Irish sample. Specifically, one microsatellite showed an allele frequency spectrum indicative of balancing selection and eight gave spectra that may support a legacy of positive selection. The use of an empirical distribution of the Ewens-Watterson test (DH/sd) to strengthen the assertion of selection and to distinguish its effects from genome-wide imprints of demographic processes confirms that, in this case, the result has not been confounded by demographic processes in the Irish population. In addition to the demonstrated utility in detecting a signature of natural selection, this approach has the particular advantage of speed. While its relative statistical power to detect selective effects, in comparison to standard tests, has yet to be elucidated, it requires considerably less laboratory effort to perform on a genomic scale, especially when compared with tests that require extensive population re-sequence data.

### The genes linked to the markers showing outlying Ewens-Watterson values

On the positive tail of the distribution, suggesting a history of balancing selection, there is 1 of 2 microsatellites (*TPSG-1*; Table 1 and Figure 3) within the TPSG1 (tryptase gamma 1; MIM:*609341) gene with four common alleles ranging in frequency from 27% to 23% (Additional data file 2). Tryptases have been implicated as mediators in the pathogenesis of asthma and other allergic and inflammatory disorders [26]. The suggestion of balancing selection in this gene is consistent with the expression of multiple tryptase isoforms, some of which are allelic variants; a common feature in genes involved in the immune response, for example, as seen in the major histocompatibility complex (MHC) of vertebrates [27].

Correlation, although limited, between LSBL and the Ewens-Watterson based statistic used here suggests that there is not complete independence between the two approaches, despite their examining different aspects of allele diversity. However, the TPSG1 outlying result is at the opposite tail to that expected under the correlation, giving stronger inference of underlying adaptive biology. Outlying results at the other tail retain a measure of complementarity given that: the correlation is weak and gives a poor empirical correspondence for outliers; the approaches draw on different aspects of population biology; and the results come from a fresh test population. We discuss two such results below.

Two microsatellites, *PRKCH-1 *and *PRKCH-2 *(Table 1 and Figure 3), within the gene PKCη (protein kinase C, eta, MIM *605437), show the most extreme negative DH/sd values. Each of these markers has one predominant allele with frequencies of 50% and 70%, respectively (Additional data file 2), consistent with an allelic configuration expected under positive selection. The majority of the other alleles have a frequency of < 10%. This shared signal was despite a distance between the markers of 79 kb (Table 1 and Figure 3); they were also in significant linkage disequilibrium (*p *< 0.001). PKC family members phosphorylate a wide variety of protein targets, are known to be involved in diverse cellular signaling pathways [28] and the protein transcribed by PKCη is involved in processes associated with several medical conditions [29-32]. Interestingly, this protein is highly expressed in the epidermis and inhibits UV-induced apoptosis of keratinocytes. It has been suggested that this mechanism prevents the excessive elimination of differentiated epidermic keratinocytes, cells that are involved in defending the skin from chemical stimulation and UV exposure [33]. A variant in this gene may have featured as part of the selected transition to light skin in the populations ancestral to Europe, although corroborating evidence would be required to assert this. Selective histories at genes related to human pigmentation have been inferred previously [34].

The next outlying marker in the negative tail of the distribution in a known gene is *IVS8CA *(Figure 3). This marker is associated with the gene CFTR (cystic fibrosis transmembrane conductance regulator; MIM:*602421), which has been well studied as mutations in it cause the autosomal recessive disorder cystic fibrosis (CF). The CFTR gene product functions as a chloride channel and controls the regulation of other transport pathways. It is also a receptor for bacterial pathogens [35,36]. There have been several hypotheses as to why CF is present at such a high level in Europe, including that of heterozygote advantage, where a single copy of the main western European disease-causing mutation (ΔF508) increases resistance to typhoid [36]. In this study, only one microsatellite, from the three examined in the gene, showed a significant deviation in the Ewens-Watterson test. Interestingly, this was also the closest to the gene region that encodes the receptor for bacterial pathogens (residues 108 to 117) [36]. The results from our study might be from selection acting on this region with an immune response function, but are unlikely to be associated with CF. The frequency of the disease in Ireland is 1/1,461 [37]. Thus, our 72 Irish samples should have about four carriers, a number too low to profoundly influence the test statistic. Interestingly, a recent analysis has stated evidence of recent selection associated with a variation (V470) that is in linkage disequilibrium with haplotypes that are not associated with the disease [38].

The remaining microsatellites (*LRRC*, *ABCD3-1*, *SYT9-1*), which have significant but less extreme values in the Ewens-Watterson test (P_S_, Table 1 and Figure 3), are associated with poorly characterized genes. However, they each appear to encode trans-membrane proteins [39-41].

## Conclusion

The results presented here provide evidence of a statistically significant deviation (P_S_) from the expectations of the neutral theory for nine microsatellite markers in the Irish population. Seven of these are associated with six genes, strengthening the likelihood that the elevated F_ST _values shown by some of the SNPs analyzed by previous studies [12] represent signatures of selection. The CFTR gene is a particularly interesting result given previous discussion of selective history and the global maximum found for the disease allele frequency within Ireland. The simple assay described here provides a means to further validate the presence of recent selection in outlier loci identified in recently published more extensive genome-wide surveys [42,43].

## Materials and methods

Microsatellites were chosen as flanking markers of the eight outlier SNPs. Perfect dinucleotide tandem repeats (according to the definition used in the University of California Santa Cruz Genome Bioinformatics Site [44]) were identified in the flanking regions of the eight outlier SNPs (Table 1) that were in, or close to, a known gene, using the UC Santa Cruz Genome Assembly (July 2003). Primers (Additional data file 1) were then designed for each microsatellite using the program GeneFisher [45]. All the microsatellite markers were typed using an ABI PRISM 377 DNA sequencer and GENESCAN software (Applied Biosystem, Foster City, California, U.S.A.).

Our sample population was composed of anonymous DNA samples from 72 unrelated Irish individuals that were made available from a previous study on the genetics of bipolar affective disorder [46], for which participants provided informed consent. The samples were previously genotyped using a commercially available set of microsatellites (ABI PRISM Linkage mapping Set-MD10), distributed throughout the whole genome.

Genotypes at each microsatellite locus were tested for Hardy-Weinberg proportions using GENEPOP [47]. Departure from the expected allele frequency distribution, a potential signature of selection, was tested using the Ewens-Watterson test. The program BOTTLENECK [22] was used to calculate at each microsatellite locus the expected heterozygosity under neutrality, according to the allelic configuration calculated with Ewens formula [23]. The program also assigned a significance value to DH/sd by completing numerous replicate simulations (P_S_, Table 1) under a specified mutation model (in this case, stepwise mutation model; 1,000 replicates per locus). A more stringent significant value (P_E_, Table 1) was also calculated directly from the empirical distribution for each screened microsatellite locus DH/sd value.

Linkage disequilibrium was calculated between linked microsatellite markers using the program ARLEQUIN [48].

A background genome-wide distribution of DH/sd values for the Irish population was calculated from 372 autosomal microsatellites (ABI-Prism MD-10; the markers on the sex chromosomes were excluded [13]) typed in the 72 individuals also used in the present study (Figure 3a). The location of each microsatellite from the genome-wide distribution, whether within a gene or not, was determined and consequently a second distribution was generated (Figure 3b). DH/sd values from the microsatellites flanking the outlier SNPs were also interpreted against this second distribution (Figure 3b) to guard against any potential confounding influences, such as the effect of purifying selection for microsatellites within genes. Pearson and Spearman correlations were performed between the distance of microsatellites in base pairs from the closest gene and their DH/sd values.

## Additional data files

The following additional data are available with the online version of this paper. Additional data file 1 is a table listing the microsatellite primer sequences and annealing temperatures. Additional data file 2 is a table listing allele frequencies at each microsatellite locus.

## Supplementary Material

Additional data file 1 Microsatellite primer sequences and annealing temperatures Click here for file

Additional data file 2 Allele frequencies at each microsatellilte locus Click here for file

## Figures and Tables

**Table 1:** SNPs, genes and microsatellites analysed in this study

SNP identity	Chromosome	Gene	Microsatellite name	Microsatellite distance from SNP (kb)	H-W	DH/sd	P_S_	P_E_
rs1009127	1	LRRC7 (leucine rich repeat 7)	*LRRC-1*	+34.6	NS	-3.6	0.007	NS
rs718830	7	CFTR (cystic fibrosis transmembrane conductance regulator)	IVS8CA*	+14.5	NS	**-4.7**	0.003	0.03
			IVS17B*	+49.2	NS	-1.1	NS	NS
			*CFTR-3*	-10.2	NS	-1.3	NS	NS
rs997929	8	TOX (thymus high mobility group box protein)	*TOX-1*	-20.4	-	-	-	-
			*TOX-2*	+22.2	NS	-1.2	NS	NS
rs726733	11	SYT9 (synaptotagmin IX)	*SYT9-1*	-7	NS	-2.8	0.019	NS
			*SYT9-2*	+40.9	NS	-1.4	NS	NS
rs1111108	14	PKCη (protein kinase C, eta)	*PRKCH-1*	+8.7	NS	**-6.3**	0.0001	0.016
			*PRKCH-2*	-70.4	NS	**-6.6**	0.0001	0.013
rs761057	16	TPSG1 (γ-triptase 1)	*TPSG-1*	-74.3	NS	**1.5**	0.001	0.003
			*TPSG-2*	+41.4	NS	-0.2	NS	NS
rs998262	5	No gene	*NG-1*	-4.6	*p *= 0.04	**-5.2**	0.0001	0.022
			*NG-2*	+31.7	NS	-3.6	0.008	NS
rs902336	1	32 kb upstream from the	*ABCD3-1*	-15.7	NS	-3.2	0.015	NS
		gene ABCD3	*ABCD3-2*	-2	-	-	-	-

'SNP identity' indicates the identification number of each SNP as annotated in dbSNP NCBI (National Center for Biotechnology Information). 'Chromosome' indicates on which chromosome the SNP is located. 'Gene' indicates the gene in which the SNP is located or the nearest gene in the case of the last SNP. 'Microsatellite name' is the name given to the microsatellites flanking the SNP; the same name can be found in Figure 3, where the microsatellites are placed in the genome-wide distribution. 'Microsatellite distance from the SNP' is the distance (in kilobase) upstream (+) or downstream (-) between the microsatellite and the SNP. These statistics are reported from analyses carried out on the microsatellite data. 'H-W' is the *p *value from the Hardy-Weinberg equilibrium test. 'DH/sd' is the observed gene diversity minus the expected heterozygosity (DH) according the Ewens-Watterson's statistic divided by the standard deviation (sd) of the gene diversity (see the text for details). 'P_S_' is the significance of the difference between observed gene diversity and expected heterozygosity resulting from the simulation carried out by the program BOTTLENECK. 'P_E_' is the significance calculated using the empirical distribution; only values < 0.05 are quoted. NS, not significant. The two microsatellites marked with an asterisk have been described previously [19,20] and no data for the two markers that were not scorable are denoted by hyphens.

## References

[B1] Hill EW, Jobling MA, Bradley DG (2000). Y-chromosome variation and Irish origins.. Nature.

[B2] McEvoy B, Richards M, Forster P, Bradley DG (2004). Longue Duree of genetic ancestry: multiple genetic marker systems and Celtic origins on the Atlantic facade of Europe.. Am J Hum Genet.

[B3] Lucotte G, Hazou S, De Braekeleer M (1995). Complete map of cystic fibrosis mutation DF508 frequencies in Western Europe and correlation between mutation frequencies and incidence of disease.. Hum Biol.

[B4] Bobadilla JL, Macek M, Fine JP, Farrell PM (2002). Cystic fibrosis: a worldwide analysis of CFTR mutations-correlation with incidence data and application to screening. Hum Mutat.

[B5] Cavalli-Sforza L, Menozzi P Piazza A (1994). The History and Geography of Human Genes.

[B6] Murphy M, McHugh B, Tighe O, Mayne P, O'Neill C, Naughten E, Croke DT (1999). Genetic basis of transferase-deficient galactosaemia in Ireland and the population history of the Irish Travellers.. Eur J Hum Genet.

[B7] Lucotte G, Mercier G (2000). Celtic origin of the C282Y mutation of hemochromatosis.. Genet Test.

[B8] DiLella AG, Kwok SCM, Ledley FD, Marvit J, Woo SLC (1986). Molecular structure and polymorphic map of the human phenylalanine hydroxylase gene.. Biochemistry.

[B9] Ronald J, Akey JM (2005). Genome-wide scans for loci under selection in humans.. Human Genomics.

[B10] Luikart G, England PR, Tallmon D, Jordan S, Taberlet P (2003). The power and promise of population genomics: from genotyping to genome typing.. Nat Rev Genet.

[B11] Bamshad M, Wooding SP (2003). Signatures of natural selection in the human genome.. Nat Rev Genet.

[B12] Akey JM, Zhang G, Zhang K, Jin L, Shriver MD (2002). Interrogating a high-density SNP map for signatures of natural selection.. Genome Res.

[B13] Payseur BA, Cutter AD, Nachman MW (2002). Searching for evidence of positive selection in the human genome using patterns of microsatellite variability.. Mol Biol Evol.

[B14] Vigouroux Y, Jaqueth JS, Matsuoka Y, Smith OS, Beavis WD, Smith JS, Doebley J (2002). Rate and pattern of mutation at microsatellite loci in maize.. Mol Biol Evol.

[B15] Kayser M, Brauer S, Stoneking M (2003). A genome scan to detect candidate regions influenced by local natural selection in human populations.. Mol Biol Evol.

[B16] Stephens JC, Schneider JA, Tanguay DA, Choi J, Acharya T, Stanley SE, Jiang R, Messer CJ, Chew A, Han JH (2001). Haplotype variation and linkage disequilibrium in 313 human genes.. Science.

[B17] Ptak SE, Preworski M (2002). Evidence for population growth in humans is confounded by fine-scale population structure.. Trends Genet.

[B18] Shriver MD, Kennedy GC, Parra EJ, Lawson HA, Sonpar V, Huang J, Akey JM, Jones KW (2004). The genomic distribution of population substructure in four populations using 8,525 autosomal SNPs.. Hum Genomics.

[B19] Morral N, Girbau E, Zielenski J, Nunes V, Casals T, Tsui LC, Estivill X (1992). Dinucleotide (CA/GT) repeat polymorphism in intron 17B of the cystic fibrosis transmembrane conductance regulator (CFTR) gene.. Hum Genet.

[B20] Zielenski J, Markiewicz D, Rininsland F, Rommens J, Tsui LC (1991). A cluster of highly polymorphic dinucleotide repeats in intron 17b of the cystic fibrosis transmembrane conductance regulator (CFTR) gene.. Am J Hum Genet.

[B21] Hosking L, Lumsdenn S, Lewis K, Yeo A, McCarthy L, Bansal A, Riley J, Purvis I, Xu CF (2004). Detection of genotyping errors by Hardy-Weinberg equilibrium testing.. Eur J Hum Genet.

[B22] Cornuet JM, Luikart G (1996). Description and power analysis of two tests for detecting recent population bottlenecks from allele frequency data.. Genetics.

[B23] Ewens WJ (1972). The sampling theory of selectively neutral alleles.. Theor Pop Biol.

[B24] Watterson GA (1978). An analysis of multi-alleleic data.. Genetics.

[B25] Rosenberg NA, Pritchard JK, Weber JL, Cann HM, Kidd KK, Zhivotovsky LA, Feldman MW (2002). Genetic structure of human populations.. Science.

[B26] Wong GW, Foster PS, Yasuda S, Qi JC, Mahalingam S, Mellor EA, Katsoulotos G, Li L, Boyce JA, Krilis SA, Stevens RL (2002). Biochemical and functional characterization of human transmembrane tryptase (TMT)/tryptase gamma. TMT is an exocytosed mast cell protease that induces airway hyperresponsiveness in vivo via an interleukin-13/interleukin-4 receptor alpha/signal transducer and activator of transcription (STAT) 6-dependent pathway.. J Biol Chem.

[B27] Garrigan D, Hedrick PW (2003). Perspective: detecting adaptive molecular polymorphism: lessons from the MHC Evolution.. Evolution Int J Org Evolution.

[B28] Parekh DB, Ziegler W, Parker PJ (2000). Multiple pathways control protein kinase C phosphorylation.. EMBO J.

[B29] Libersan D, Merhi Y (2003). Platelet P-selectin expression: requirement for protein kinase C, but not protein tyrosine kinase or phosphoinositide 3-kinase.. Thromb Haemost.

[B30] Brenner W, Farber G, Herget T, Wiesner C, Hengstler JG, Thuroff JW (2003). Protein kinase C eta is associated with progression of renal cell carcinoma (RCC).. Anticancer Res.

[B31] Vattemi G, Tonin P, Mora M, Filosto M, Morandi L, Savio C, Dal Pra I, Rizzuto N, Tomelleri G (2004). Expression of protein kinase C isoforms and interleukin-1beta in myofibrillar myopathy.. Neurology.

[B32] Aeder SE, Martin PM, Soh JW, Hussaini IM (2004). PKC-eta mediates glioblastoma cell proliferation through the Akt and mTOR signaling pathways.. Oncogene.

[B33] Matsumura M, Tanaka N, Kuroki T, Ichihashi M, Ohba M (2003). The eta isoform of protein kinase C inhibits UV-induced activation of caspase-3 in normal human keratinocytes.. Biochem Biophys Res Commun.

[B34] Lamason RL, Mohideen MA, Mest JR, Wong AC, Norton HL, Aros MC, Jurynec MJ, Mao X, Humphreville VR, Humbert JE (2005). SLC24A5, a putative cation exchanger, affects pigmentation in zebrafish and humans.. Science.

[B35] Pier GB, Grout M, Zaidi T, Meluleni G, Mueschenborn SS, Banting G, Ratcliff R, Evans MJ, Colledge WH (1998). Salmonella typhi uses CFTR to enter intestinal epithelial cells.. Nature.

[B36] Pier GB (2000). Role of the cystic fibrosis transmembrane conductance regulator in innate immunity to Pseudomonas aeruginosa infections.. Proc Natl Acad Sci USA.

[B37] Devaney J, Glennon M, Farrell G, Ruttledge M, Smith T, Houghton JA, Maher M (2003). Cystic fibrosis mutation frequencies in an Irish population.. Clinical Genet.

[B38] Pompei F, Ciminelli BM, Bombieri C, Ciccacci C, Koudova M, Giorgi S, Belpinati F, Begnini A, Cerny M, Des Georges M (2006). Haplotype block structure study of the CFTR gene. Most variants are associated with the M470 allele in several European populations.. Eur J Hum Genet.

[B39] Fukuda M, Kowalchyk JA, Zhang X, Martin TF, Mikoshiba K (2002). Synaptotagmin IX regulates Ca2+-dependent secretion in PC12 cells.. J Biol Chem.

[B40] Izawa I, Nishizawa M, Ohtakara K, Inagaki M (2002). Densin-180 interacts with delta-catenin/neural plakophilin-related armadillo repeat protein at synapses.. J Biol Chem.

[B41] Liu LX, Janvier K, Berteaux-Lecellier V, Cartier N, Benarous R, Aubourg P (1999). Homo- and heterodimerization of peroxisomal ATP-binding cassette half-transporters.. J Biol Chem.

[B42] Hinds DA, Stuve LL, Nilsen GB, Halperin E, Eskinm E, Ballinger DG, Frazer KA, Cox DR (2005). Whole-genome patterns of common DNA variation in three human populations.. Science.

[B43] Altshuler D, Brooks LD, Chakravarti A, Collins FS, Daly MJ, Donnelly P, International HapMap Consortium (2005). A haplotype map of the human genome.. Nature.

[B44] The University of California Santa Cruz Genome Bioinformatics Site. http://genome.ucsc.edu/index.html.

[B45] GeneFisher. http://bibiserv.techfak.uni-bielefeld.de/genefisher/help/wwwgfdoc.html.

[B46] Bennett P, Segurado R, Jones I, Bort S, McCandless F, Lambert D, Heron J, Comerford C, Middle F, Corvin A (2002). The Wellcome trust UK-Irish bipolar affective disorder sibling-pair genome screen: first stage report.. Mol Psychiatry.

[B47] Raymond M, Rousset F (1995). GENEPOP: population genetics software for exact test and ecumenicism.. J Hered.

[B48] Schneider S, Roessli D, Excoffier L (2000). ARLEQUIN Version 200: a Software for Population Genetics Data Analysis.

