# Supplementary material for: A genome-wide approach to identify genetic loci with a signature of natural selection in the Irish population
Source: Genome Biol. 2006 Aug 11;7(8):R74. doi: 10.1186/gb-2006-7-8-r74 (PMC1779589; doi:10.1186/gb-2006-7-8-r74)
Supplement: Additional data file 1 — Microsatellite primer sequences and annealing temperatures [file gb-2006-7-8-r74-S1.doc]

**Supplementary material Table A**

Microsatellites primer sequences and annealing temperatures.

| **Marker Name** | **Primer sequences** | **Annealing T°** | **most common allele** |
| --- | --- | --- | --- |
| *PRKCH-1* | Fw- CATCATCTTGGAGCCATGAGGCAA  Rev- AGATACAAAGAGCAGCACGAGA | 58°C  (2mM Mg^2++^) | 232bp |
| *PRKCH-2* | Fw-AAGATTGCTGGCTTTCTCCA  Rev-GTGTTCCAGAGTCATTCTTCCA | 58°C  (2mM Mg^2++^) | 249bp |
| *TPSG-1* | Fw-AAAGGGAGGCGGGATGTACACA  Rev-GGCCTCTGTTCTTTGAAAGGCAA | 58°C  (1.5mM Mg^2++^) | 223bp |
| *TPSG-2* | Fw-TGCAGTGAGCCAAGGTCATACCA  Rev-TTAAGGGACCAAGAGTACTGCTGA | 58°C  (1.5mM Mg^2++^) | 141bp |
| *SYT9-1* | Fw-ATCCTCAAGTTCTCCATGGGCTGA  Rev-AATATGTCTTGGGTGAGGTCCAGA | 58°C  (1.5mM Mg^2++^) | 258bp |
| *SYT9-2** | Fw-AGAGATGGTTGCACAACACTGTGA  Rev-AACCCAGCCCTTGCTGTATGGA | 58°C  (1.5mM Mg^2++^) | 327bp |
| *ABCD-1* | Fw-CGGAAGTTCCAGTGAGCTGA  Rev-AAACTGGTCCAGTAAGTGGTGA | - | - |
| *ABCD-2* | Fw-AGCTTCCAGCAAACACTTCATCCA  Rev-AGGGAACCATTTCATTCCCATCCA | 58°C  (1.5mM Mg^2++^) | 365bp |
| *ng-1* | Fw-CTGGTTGGCCTGATTTTCCCTGA  Rev-TTGACCTAAAGGTGGGATACGTGA | 60°C  (1.5mM Mg^2++^) | 321bp |
| *ng-2* | Fw-GGGCAATGGAAGTGGTTTC  Rev-ACCTGGGTCCTGCAATAG | 55°C  (1.5mM Mg^2++^) | 311bp |
| *TOX-1* | Fw-CTGAGACAGGAAAATCGCTTGCA  Rev-ATATACCTTCTAGGCTGCTCCTGA | - | - |
| *TOX-2* | Fw-TCAAAGCCTCCATTGGCCCTCA  Rev-CAGAGCAACACAGTTTGGTTCAGA | 59°C  (1.5mM Mg^2++^) | 373bp |
| *KIAA* | Fw-ATGAGAACATTGCTACCCACCA  Rev-GTAGATGAAGGAGGCTGGGACTGA | 58°C  (1.5mM Mg^2++^) | 249bp |
| *IVS8CA* | See Morral et al.1992 | 52°C  (1.5mM Mg^2++^) | 176bp |
| IVS17Bta | See Zieliensky et al. 1991 | 52°C  (1.5mM Mg^2++^) | 200bp,246bp |
| CFTR-3 | Fw-CTACAAAGTCACAGGAAGCTCA  Rev-CAACTGGACCAAACAAACCAGA | 58°C  (1.5mM Mg^2++^) | 234bp |
