# Supplementary material for: A genome-wide approach to identify genetic loci with a signature of natural selection in the Irish population
Source: Genome Biol. 2006 Aug 11;7(8):R74. doi: 10.1186/gb-2006-7-8-r74 (PMC1779589; doi:10.1186/gb-2006-7-8-r74)
Supplement: Additional data file 2 — Allele frequencies at each microsatellilte locus [file gb-2006-7-8-r74-S2.doc]

**Supplementary material Table B**

Allele frequencies at each microsatellilte locus

| Locus | Allele size in bp | Frequency |
| --- | --- | --- |
| *CFTR-3* | 222  234  240  242 | 18.33  72.50  8.33  0.83 |
| *IVS8CA* | 172  174  176  178  180  186  190 | 3.85  3.08  65.38  16.15  2.31  2.31  6.92 |
| *IVS17bTA* | 200  226  234  240  244  246  248  250  252  254  256  260  262  270  274  276  278  280  288  292  294 | 15.63  1.56  3.91  1.56  8.59  15.63  10.94  8.59  1.56  0.78  8.59  4.69  0.78  0.78  0.78  7.03  3.91  0.78  0.78  1.56  1.56 |
| *Ng-2* | 305  309  311  313  315  317  319 | 3.03  13.64  64.39  8.33  3.03  3.03  4.55 |
| *Ng-1* | 303  311  315  317  319  321  325  327  329  331  333  335 | 1.52  0.76  0.76  3.03  1.52  44.70  17.42  18.94  6.82  0.76  3.03  0.76 |
| *LRRC-1* | 235  239  241  245  247  249  251  253  255 | 6.25  2.34  0.78  0.78  4.69  51.56  15.63  17.19  0.78 |
| *TOX-2* | 365  367  369  371  373  375  377  379 | 0.82  4.92  2.46  17.21  36.07  31.15  4.10  3.28 |
| *TOX-1* | 313  333  337  339  351  355  359  361  363  365  367  371  373  375  377 | 0.91  0.91  5.45  0.91  0.91  1.82  2.73  4.55  1.82  48.18  17.27  4.55  1.82  6.36  1.82 |
| *ABCD-1* | 363  365  367  369  371 | 19.35  75.81  0.81  3.23  0.81 |
| *TPSG-2* | 141  143  145  147  149 | 52.63  22.81  4.39  14.04  6.14 |
| *PRKCH-2* | 239  243  249  253  255  257  259 | 0.79  3.17  76.98  13.49  1.59  3.17  0.79 |
| *SYT9-1* | 250  252  254  256  258  259  262 | 0.77  53.85  30.00  3.85  10.00  0.77  0.77 |
| *PRKCH-1* | 216  220  224  226  228  230  232  233  234  236  238 | 1.61  4.84  0.81  2.42  4.84  0.81  53.23  0.81  18.55  9.68  2.42 |
| *SYT9-2* | 319  325  327  329  331  333  335 | 12.10  1.61  50.00  14.52  1.61  17.74  2.42 |
| *TPSG-1* | 217  219  221  223 | 24.55  24.55  23.64  27.27 |
